# *Pistacia chinensis*: A Potent Ameliorator of CCl_4_ Induced Lung and Thyroid Toxicity in Rat Model

**DOI:** 10.1155/2014/192906

**Published:** 2014-08-11

**Authors:** Kiran Naz, Muhammad Rashid Khan, Naseer Ali Shah, Saadia Sattar, Farah Noureen, Madeeha Latif Awan

**Affiliations:** ^1^Department of Biochemistry, Faculty of Biological Sciences, Quaid-i-Azam University, Islamabad 45320, Pakistan; ^2^Department of Biosciences, COMSATS Institute of Information Technology, Islamabad, Pakistan

## Abstract

In the current study protective effect of ethanol extract of *Pistacia chinensis* bark (PCEB) was investigated in rats against CCl_4_ induced lung and thyroid injuries. PCEB dose dependently inhibited the rise of thiobarbituric acid-reactive substances, hydrogen peroxide, nitrite, and protein content and restored the levels of antioxidant enzymes, that is, catalase, peroxidase, superoxide dismutase, glutathione-S-transferase, glutathione reductase, glutathione peroxidase, *γ*-glutamyl transpeptidase, and quinone reductase in both lung and thyroid tissues of CCl_4_ treated rats. Decrease in number of leukocytes, neutrophils, and hemoglobin and T_3_ and T_4_ content as well as increase in monocytes, eosinophils, and lymphocytes count with CCl_4_ were restored to normal level with PCEB treatment. Histological study of CCl_4_ treated rats showed various lung injuries like rupture of alveolar walls and bronchioles, aggregation of fibroblasts, and disorganized Clara cells. Similarly, histology of CCl_4_ treated thyroid tissues displayed damaged thyroid follicles, hypertrophy, and colloidal depletion. However, PCEB exhibited protective behaviour for lungs and thyroid, with improved histological structure in a dose dependant manner. Presence of three known phenolic compounds, that is, rutin, tannin, and gallic acid, and three unknown compounds was verified in thin layer chromatographic assessment of PCEB. In conclusion, *P. chinensis* exhibited antioxidant activity by the presence of free radical quenching constituents.

## 1. Introduction

Free radicals are the major by-products produced by the cells of aerobic organisms and can start the autocatalytic reactions and spread the chain of damage by reacting with molecules and converting them into free radicals. Free radicals are mainly produced from these sources in the body: ubisemiquinone in the mitochondrial membrane, xanthine oxidase of endothelial cells, and myeloperoxidase and NADPH oxidase of neutrophils. But, xanthine oxidase and respiratory chain of mitochondria are the major sources of reactive oxygen species (ROS) [1]. Free radicals are extremely unstable and become stable by pairing an outer shell electron with biomolecules, for example, lipids, proteins, and DNA. At high concentrations, free radicals can cause damage to various cell structures, comprising proteins and nucleic acids, together with lipid peroxidation [2, 3]. These injuries and damages are the major contributions towards aging, cardiovascular diseases, atherosclerosis, cancer, and inflammatory diseases [4]. Living cells contain a defensive system of antioxidants against the ROS which avoids their unnecessary production and inactivate them. Various antioxidants have been reported to protect the body from the damaging effects of oxidative stress. Nowadays, research has been increased in the area of finding novel natural antioxidants due to low side effects in comparison to synthetic antioxidants [5].

Carbon tetrachloride (CCl_4_) is a commonly used xenobiotic to induce toxicity in animal models. It is well known as a hepatotoxin [6], nephrotoxin [7], and pulmonary toxin [8] and also produces injuries in other organs. It has been demonstrated that oxidative stress caused by CCl_4_ is due to production of reactive free radicals such as hydrogen peroxides, hydroxyl radicals, super oxides, peroxy nitrite, and many other radicals [9].

The best source to get a range of novel herbal drugs is medicinal plants. Medicinal plants are local heritage with universal importance. Various medicinal agents have been derived from the natural sources; among them a lot of drugs are formulated due to their utilization in traditional medicine. Medicinal plants comprise vast range of organic substances which are capable to prevent most of the diseases related to oxidative stress [10] and these plants are commonly used in the folk medicine for treatment of many diseases [11]. Genus* Pistacia* is comprised of 11 species and belongs to the family “Anacardiaceae.”* Pistacia* is traditionally used in the treatment of cough, asthma, fever, and respiratory disorders [12]. In order to discover new economical, nontoxic, and natural antioxidants from* P. chinensis* and to observe the effect of its ethanol bark extract on tissue related animal health, CCl_4_ was used as toxicant for lungs and thyroid gland in the present study.

## 2. Materials and Methods

### 2.1. Chemicals

CCl_4_, reduced glutathione (GSH), glutathione reductase, *γ* *-*glutamyl p-nitroanilide, bovine serum albumin (BSA), 1,2-dithio-bis nitro benzoic acid (DTNB), 1-chloro-2,4-dinitrobenzene (CDNB), reduced nicotinamide adenine dinucleotide phosphate (NADPH), flavin adenine dinucleotide (FAD), 2,6-dichlorophenolindophenol, thiobarbituric acid (TBA), picric acid, potassium iodide, sodium tartrate, copper sulphate, bromo cresol green, hydrogen peroxide solution, phenazine methosulphate, glycylglycine, magnesium chloride, guaiacol, sulfosalicylic acid, sodium azide, reduced nicotinamide adenine dinucleotide (NADH), sodium hydroxide, and trichloroacetic acid (TCA) were purchased from Sigma Chemicals Co. USA.

### 2.2. Plant Collection

The barks of* P. chinensis* were collected from the campus of Quaid-i-Azam University, Islamabad, Pakistan, and were recognized by their local names and then confirmed by Prof. Dr. Mir Ajab Khan, Department of Plant Sciences, Quaid-i-Azam University, Islamabad. Voucher specimen with Accession Number 27840 was deposited at the Herbarium, Quaid-i-Azam University, Islamabad. Plant sample (2 kg) was washed with distilled water and dried at room temperature in a shade for more than two weeks and grinded using electrical grinder.

#### 2.2.1. Preparation of Extract

For the preparation of ethanol extract of* P. chinensis* 4 litres of 80% ethanol was added to the 2 kg of powdered bark and sonicated for 2 h at 45°C. The extract was filtered by using Whatman filter paper number 45 after a week. Rotary vacuum evaporator was used to evaporate the solvent from filtrate and to obtain ethanol extract of* P. chinensis* (PCEB).

### 2.3. Phytochemical Analysis

#### 2.3.1. Total Phenolic Content Estimation

Spectrophotometric method [13] was used with minor modifications to determine total phenolic content. In short, 200 *μ*L of the sample (1 mg/mL) was mixed with 1 mL of the 1 : 10 Folin-Ciocalteu's reagent. To the mixture 2.5 mL of 7% Na_2_CO_3_ was added after 5 min. The mixture was incubated at 23°C in the dark for 90 min. Absorbance was recorded at 765 nm. Total phenolic content was calculated from calibration curve of gallic acid. Estimation of total phenolic content was recorded in triplicate and presented as mg of gallic acid equivalents (GAE) per g of dry sample.

#### 2.3.2. Total Flavonoid Content Estimation

In a test tube, 0.3 mL of sample (1 mg/mL), 3.4 mL of 30% methanol, 0.15 mL of 0.5 M NaNO_2_, and 0.1 mL of 0.3 M AlCl_3_ *·*6H_2_O were thoroughly mixed. After 5 min, 1 mL of 1 M NaOH was added and mixed well. Absorbance was measured at 506 nm against the reagent blank. Total flavonoid content was estimated by using a calibration curve of rutin and expressed as mg rutin equivalents per g of dry sample [14].

### 2.4. Thin Layer Chromatography

Thin layer chromatography was carried out for the presence of phenolics in the extract. Extract (50 mg) was dissolved in 1 mL of methanol. Precoated TLC plates of 20 × 20 cm and 0.25 mm thick were used in the experiment. For the activation of silica, plates were heated at 100°C for 45 min by placing them in oven. On the lower surface of plate, a line of 1 cm was drawn and 6 *μ*L of extract was plotted. After drying, the plates were run in the mixture of* n*-butanol, acetic acid, and water in a ratio of 4 : 1 : 5. Plates were removed when solvent reached the end of TLC. Solvent front was marked and plates were sprayed with ethanolic 2-aminoethyle diphenyl borinate solution (1%) followed by 5% solution of ethanolic polyethylene glycol. Flavonoids were detected by observing its characteristic colors under UV light at 360 nm. Different bands were observed and their *R*_*f*_ values were calculated by the following formula:

(1) $$\begin{array}{c} R_{f}\;\;\text{value}=\frac{\text{Distance}\;\;\text{covered}\;\;\text{by}\;\;\text{band}}{\text{Distance}\;\;\text{covered}\;\;\text{by}\;\;\text{solvent}}. \end{array}$$

### 2.5. *In Vivo* Studies

For* in vivo* studies CCl_4_ was used as a source of free radicals to produce toxicity in lungs and thyroid gland of rat model. Lungs and thyroid tissues were investigated at biochemical and histological level.

#### 2.5.1. Experimental Design

Shah et al. [14] protocol was followed for the experimental design. Male Sprague Dawley rats (160–210 g) of seven weeks old were used as animal model in this study. They were maintained in cages at room temperature of 25 ± 3°C with a 12 h light/dark cycle and free access to water and feed. The study protocol was approved (No. 0246) by the ethical committee of Quaid-i-Azam University, Islamabad, Pakistan, for laboratory animal care and experimentation.

Forty-two male rats were randomly distributed into 7 groups (6 rats/group). Group I remained untreated. Group II was treated with 30% CCl_4_ in olive oil (1 mL/kg b.w.), intraperitoneally. Groups III, IV, and V were orally given silymarin (100 mg/kg b.w.), PCEB (200 mg/kg rat b.w.), and PCEB (400 mg/kg b.w.), respectively, after one hour of 30% CCl_4_ injection. Groups VI and VII were given only PCEB (400 mg/kg b.w.) and olive oil (1 mL/kg b.w.), respectively, by oral route. Olive oil was used as vehicle for extract and silymarin treatment. Experiment was comprised of 60 days and treatments were given on alternative day.

#### 2.5.2. Animal Dissection

After last treatment, rats were unfed for 24 h. Chloroform was used to anesthetize animals and then dissected the animals from ventral side of the body. Blood was collected by piercing heart. Blood was collected in two types of tubes; that is, for serum analysis it was stored in small falcon tubes which was then centrifuged to obtain serum; the rest of the blood was collected in EDTA containing tubes for whole blood analysis. From the dissected animal, lungs and thyroid glands were removed and placed in saline solution. For histology, half of the lung and thyroid was stored in 10% formalin solution while the half was stored in liquid Nitrogen at −70°C for antioxidant enzymes and tissue stress marker examination.

#### 2.5.3. Serum Analysis

After dissection of animals, blood was collected in falcon tubes and centrifuged at 4000 rpm for 20 min at 4°C, after 30 minutes, to collect the serum samples. Total protein, albumin, and globulin, in serum samples, were estimated with the help of AMP diagnostics company kits, while T_3_ and T_4_ were analysed by using MicroLISA kits.

*(1) Total Protein Estimation*. Estimation of total protein is based on Biuret reaction which measures the amount of colored complex formed when protein reacts with alkaline solution in the presence of copper salt. An aliquot of 10 *μ*L of serum sample was added to 1 mL reagent (NaOH 53 mM, CuSO_4_ 6 mM, potassium iodide 6 mM, potassium sodium tartarate 21 mM). After incubation of 10 min at 37°C, optical density was measured at 550 nm spectrophotometrically. Distilled water plus reagent was used as a blank and albumin was used as a standard. For the calculation of total protein the following formula was used:

(2) $$\begin{array}{c} \text{Total}\;\;\text{Protein}\;\;\text{Concentration}\;\;\left(\frac{\text{g}}{\text{dL}}\right)=\frac{\text{Abs}\;\;\text{Sample}}{\text{Abs}\;\;\text{Standard}}\times n, \end{array}$$

Where *n* is concentration of standard (mg/dL).

*(2) Albumin Estimation*. Albumin estimation is based on the principle that in acidic medium (pH 3.8) albumin reacts with BCG (bromocresol green) to produce a colored complex, which is measured spectrophotometrically. An aliquot of 10 *μ*L of serum sample was added to 1.5 mL reagent (bromocresol green 0.25 mM, citrate buffer 100 mM, Triton X-100 10 g/L). Optical density was measured at 628 nm spectrophotometrically. Distilled water plus reagent was used as a blank and albumin (40 g/L) was used as a standard. For the calculation of albumin the following formula is used:

(3) $$\begin{array}{c} \text{Albumin}\;\;\text{Concentration}\;\;\left(\frac{\text{g}}{\text{dL}}\right)=\frac{\text{Abs}\;\;\text{Sample}}{\text{Abs}\;\;\text{Standard}}\times n, \end{array}$$

Where *n* is concentration of standard (mg/dL).

*(3) Globulin Estimation*. The following formula was used for globulin estimation in serum and urine samples:

(4) $$\begin{array}{c} \text{Globulin}\;\;\text{concentration}\;\;\left(\frac{\text{g}}{\text{dL}}\right)=\text{Total}\;\;\text{protein}-\text{Albumin}. \end{array}$$

#### 2.5.4. Triiodothyronine (T_3_) or Thyroxine (T_4_) Estimation

For the estimation of triiodothyronine (T_3_) or thyroxine (T_4_) specific amount of anti-T_3_ or anti-T_4_ antibody is coated on the microtiter wells. A measured amount of serum and a constant amount of T_3_ or T_4_ conjugated with horseradish peroxidase are added to the microtiter wells. During incubation, T_3_ or T_4_ present in the serum and conjugated T_3_ or T_4_ compete for the limited binding sites on the anti-T_3_ or anti-T_4_ antibody. After 60 min incubation, the wells are washed to remove unbound T_3_ or T_4_ conjugate. A solution of TMB substrate is added which results in formation of blue color. The color development is stopped by addition of 2 N HCl and absorbance is measured spectrophotometrically at 450 nm. The intensity of color is directly proportional to the amount of enzyme present and inversely related to the unlabeled T_3_ or T_4_ in the sample. Series of standards are run in the same way to quantify the concentration of T_3_ or T_4_ in sample. Standards, samples, and controls (50 *μ*L) are added into appropriate wells and 100 *μ*L of enzyme conjugate is also added. After mixing the microtiter plate is incubated at room temperature for 60 minutes. After incubation wells are washed 5 times with 1x washing buffer and 100 *μ*L of TMB substrate is added in each well. After 20 min incubation 100 *μ*L of stop solution is added and optical density is measured at 450 nm.

#### 2.5.5. Haematological Analysis

Anticoagulated blood samples were used for the determination of total leukocyte count, neutrophils, lymphocytes, eosinophils, monocytes, and haemoglobin level by using cell DYN ruby automated 5-part hematology analyzer (Abbott diagnostics, Germany).

#### 2.5.6. Antioxidant Enzymes Estimation

Tissue homogenate was prepared by homogenizing 100 mg of lung and thyroid tissue in 1 mL of 100 mM potassium phosphate buffer containing 1 mM EDTA at pH 7.4. Supernatant was collected in clean falcon tubes after centrifugation for 30 min at 12000 ×g at 4°C and was used for further analysis. The following assays were carried out to analyze the pharmacological activity against the toxicity induced with CCl_4_ in rats.

*(1) Catalase (CAT) Activity*. Measurement of CAT activity is based on the methodology of Shah et al. [14], which relies on decomposition of H_2_O_2_. An aliquot of 25 *μ*L of tissue homogenate was added to 100 *μ*L of 10 mM H_2_O_2_ and 625 *μ*L of 5 mM EDTA buffer (pH 8.0). The disappearance of H_2_O_2_ in the reaction mixture by catalase was measured spectrophotometrically at 230 nm. CAT activity was expressed as U/mg protein.

*(2) Peroxidase (POD) Activity*. POD activity was measured spectrophotometrically by the method of Khan and Ahmed [15]. An aliquot of 25 *μ*L of tissue homogenate was added to 1 mL of pyrogallol solution and 125 *μ*L of H_2_O_2_ was added and mixed. After one minute change in absorbance was measured at 430 nm. Change in absorbance/min is defined as one unit POD activity. 

*(3) Super Oxide Dismutase (SOD) Activity*. Measurement of SOD activity was carried out according to Kakkar et al. [16] by using sodium pyrophosphate buffer and phenazine methosulphate. Tissue homogenate was centrifuged for 10 min at 1500 ×g followed by 10000 ×g for 15 min. Supernatant was collected and used to determine SOD activity. An aliquot containing 150 *μ*L of supernatant was added to 600 *μ*L of 0.052 mM sodium pyrophosphate buffer (pH 7.0) and 50 *μ*L of 186 *μ*M phenazine methosulphate. 100 *μ*L of 780 *μ*M NADH was added to start enzymatic reaction. Addition of 500 *μ*L of glacial acetic acid after 1 minute stops the reaction. Optical density was determined at 560 nm to measure the color intensity. Results are expressed in units/mg protein. 

*(4) Gamma Glutamyl Transpeptidase (*γ*-GT) Activity*. To determine the activity of *γ*-GT, methodology of Fossati and Prencipe [17] was followed. Two reagent solutions were prepared in Tris buffer (50 mM/L, pH 8 at 37°C). Reagent 1 contains, per litre of Tris, 116 mM of glycyl glycine, 3.6 mM of 2, 5-dimethylphenol, 12 kU of ascorbate oxidase, and 5 g of Triton X-100 surfactant. Reagent 2 contains 20 mM of glycylglycine and 24 mM of *γ*-glu-DBHA per litre of Tris buffer. An aliquot of 50 *μ*L of tissue homogenate was added to mixture containing 1 mL of reagent 1 and 200 *μ*L of reagent 2. Enzyme activity was measured at 37°C by recording the absorbance changes for 180 s after a 2 min lag phase at 620 nm. 

*(5) Reduced Glutathione (GSH) Activity*. GSH activity was measured according to method of Jollow et al. [18]. 500 *μ*L of tissue homogenate was precipitated by addition of 500 *μ*L of sulfosalicylic acid (4%). After 1 hour incubation at 4°C, samples were centrifuged for 20 min at 1200 ×g. 33 *μ*L of supernatant was collected and added to aliquots containing 900 *μ*L of 0.1 M potassium phosphate buffer (pH 7.4) and 66 *μ*L of 100 mM DTNB. Reduced glutathione reacts with DTNB and forms a yellow colored complex. Absorption was measured at 412 nm. *μ*M GSH/g tissue represents GSH activity. 

*(6) Glutathione Peroxidase (GSH-Px) Activity*. Glutathione peroxidase (GSH-Px) was determined colorimetrically according to the method of Ozdemir et al. [19] using NADPH-coupled reduction of GSSG catalysed by Glutathione reductase which can be measured at 340 nm. Using molar coefficient of 6.23 × 10^3^/M/cm, GSH-Px activity was determined as amount of NADPH oxidized/min/mg protein. 

*(7) Quinone Reductase (QR) Activity*. Quinone reductase activity is measured by a method of Benson et al. [20], which is based on reduction of dichloro phenol indophenol complex. An aliquot of 33.3 *μ*L of tissue homogenate was added to 233 *μ*L of bovine serum albumin, 6.6 *μ*L of 0.1 mM NADPH, 33.3 *μ*L of 50 mM FAD, and 710 *μ*L of 25 mM Tris-HCL buffer (pH 7.4). Optical density was measured at 600 nm. Using molar extinction coefficient of 2.11 × 10^4^/M/cm, QR activity was determined as nmoles of DCPIP reduced/min/mg protein. 

*(8) Glutathione Reductase (GSR) Activity*. GSR activity was determined by using NADPH as substrate [21]. An aliquot of 50 *μ*L of tissue homogenate was added to 50 *μ*L of 0.5 mM EDTA, 50 *μ*L of 0.1 mM NADPH, 25 *μ*L of 1 mM oxidized glutathione, and 825 *μ*L of 0.1 M sodium phosphate buffer (pH 7.6). With the help of spectrophotometer, decomposition of NADPH is measured at 340 nm at 25°C, using molar extinction coefficient of 6.23 × 10^3^/M/cm. GSR activity was determined as amount of NADPH oxidized/min/mg protein. 

*(9) Glutathione-S-Transferase (GST) Activity*. GST assay was based on the formation of CDNB conjugate [22]. An aliquot of 150 *μ*L of tissue homogenate was added to 100 *μ*L of 1 mM reduced glutathione, 12.5 *μ*L of 1 mM CDNB, and 720 *μ*L of sodium phosphate buffer. Optical density was measured at 340 nm, using a molar coefficient of 9.61 × 10^3^/M/cm. GST activity was measured as amount of CDNB conjugate formed/min/mg protein. 

*(10) Lipid Peroxidation Assay (TBARS)*. Using TBA, TBARS (thiobarbituric acid reactive substances) were measured in the tissue homogenate [23]. An aliquot of 100 *μ*L of tissue homogenate was added to 100 *μ*L of 100 mM ascorbic acid, 10 *μ*L of 100 mM FeCl_3_, and 290 *μ*L of sodium phosphate buffer (pH 7.4). Reaction solution was incubated for 1 hour in a shaking water bath at 37°C. Addition of 500 *μ*L of 10% TCA stopped the reaction. Place reaction tubes were placed in boiling water bath for 15 minutes after adding 500 *μ*L of 0.67% TBA. After 15 min tubes were shifted on a crushed ice for 5 minutes and centrifuged for 10 minutes at 2500 ×g. Optical density of supernatant was measured at 535 nm to determine the amount of TBARS formed. Using molar extinction coefficient of 1.560 × 10^5^/M/cm, lipid per oxidation activity was measured as an amount of TBARS formed/min/mg tissue. 

*(11) Nitrite/Nitrate Assay*. Nitrite/nitrate (NO) was assayed calorimetrically in tissue homogenate by using methodology of Berkels et al. [24]. Promega's Griess reagent system is based on the chemical reaction between sulphanilamide and N-1-naphthylethylenediamine dihydrochloride under acidic condition (phosphoric acid) to give bright reddish-purple color azocompound which can be measured at 540 nm spectrophotometrically. Using standard curve of sodium nitrite, nitrite concentration in tissue samples was calculated. 

*(12) H*_*2*_ *O*_*2*_ *Assay*. The methodology of Pick and Keisari [25] was adopted to determine the H_2_O_2_-mediated horseradish peroxidase-dependent oxidation of phenol red. An aliquot of 100 *μ*L of tissue homogenate was added to 100 *μ*L of 0.28 nM phenol red, 250 *μ*L of 5.5 nM dextrose, 8 units of horse radish peroxidase, and 500 *μ*L of 0.05 M phosphate buffer (pH 7.0) and incubated at room temperature for 1 hour. Reaction was stopped by the addition of 100 *μ*L of 10 N NaOH and then tubes were centrifuged for 10 min at 800 ×g. Supernatant was collected and absorbance was measured at 610 nm using reagent as blank. The quantity of H_2_O_2_ produced was expressed as nM H_2_O_2_/min/mg tissue based on the standard curve of H_2_O_2_ oxidized phenol red. 

*(13) Tissue Protein Estimation*. Total amount of soluble proteins in tissue homogenate was determined by method of Lowry et al. [26]. To the tissue homogenate 300 *μ*L of 0.1 M potassium phosphate buffer (pH 7.0) was added in order to dilute the tissue sample. To this mixture 1 mL of alkaline copper solution was added and kept at room temperature. After 10 min of incubation, 100 *μ*L of Folin-Ciocalteau phenol reagent was added. Reaction tubes containing test mixture were then vortexed and again incubated at 37°C for 30 minutes. At 650 nm optical density was measured spectrophotometrically. Total soluble proteins of tissue samples were then determined using standard curve of bovine serum albumin.

#### 2.5.7. Histopathological Study

For histopathological studies, lung and thyroid tissues were fixed in 10% formalin and embedded in paraffin, sectioned at 4 *μ*m, and subsequently stained with hematoxylin/eosin. Slides were photographed and studied under light microscope at 10x and 40x.

### 2.6. Statistical Analysis

The values were expressed as means ± standard deviation (SD) of six observations in each group. Multiple comparison test (Tukey HSD) by Statistix 8.1 software at 0.05% level of probability among different groups was performed and expressed in the form of homogenous group.

## 3. Results

Many medicinal characteristics are found in the medicinal plants and are commonly used in herbal drug. As synthetic drugs have many side effects, scientists had moved towards natural medicines. Nowadays, all over the world scientists are exploring the scientific basis of effectiveness of traditional medicine and in this context many herbal drugs have been analyzed and their phytochemicals have been isolated and presented to the pharmaceutical industries for drug formulation.

### 3.1. Estimation of Total Flavonoid and Phenolic Contents

Phenolics and flavonoids are most potent constituent of plants that is responsible for the important antioxidant behavior. Therefore their quantification is an important step to estimate its quantity. Total flavonoids contents were estimated as 325 ± 10.5 mg rutin/g of dry extract while total phenolic content was estimated as 226 ± 11.5 mg gallic acid equivalent/g of dry extract.

### 3.2. Thin Layer Chromatography

For visualization of flavonoids and phenolics present in ethanol bark extract of* P. chinensis*, thin layer chromatography was used. Six standard compounds were also run to optimize their *R*_*f*_ values. These standards include gallic acid (*R*_*f*_ = 0.81, fluorescent blue), ascorbic acid (*R*_*f*_ = 0.50, dark purple), catechin (*R*_*f*_ = 0.87, dark brown), kaempferol (*R*_*f*_ = 0.75, olive green), rutin (*R*_*f*_ = 0.57, yellow), and tannin (*R*_*f*_ = 0.78, light blue).

Table 1 describes the color and thin layer chromatographic *R*_*f*_ values of the ethanol extract of* P. chinensis* bark. The extract was found to contain rutin, tannin, gallic acid, and unknown compounds with *R*_*f*_ values of 0.18, 0.43, and 0.75.

### 3.3. *In Vivo* Studies

The present experiment was carried out to determine the protective role of* P. chinensis* extract against CCl_4_ induced lung and thyroid toxicity at biochemical and histological level in rats.

#### 3.3.1. Estimation of CCl_4_ Toxicity on Serum Protein, Albumin, and Globulin

**Figure 1:**
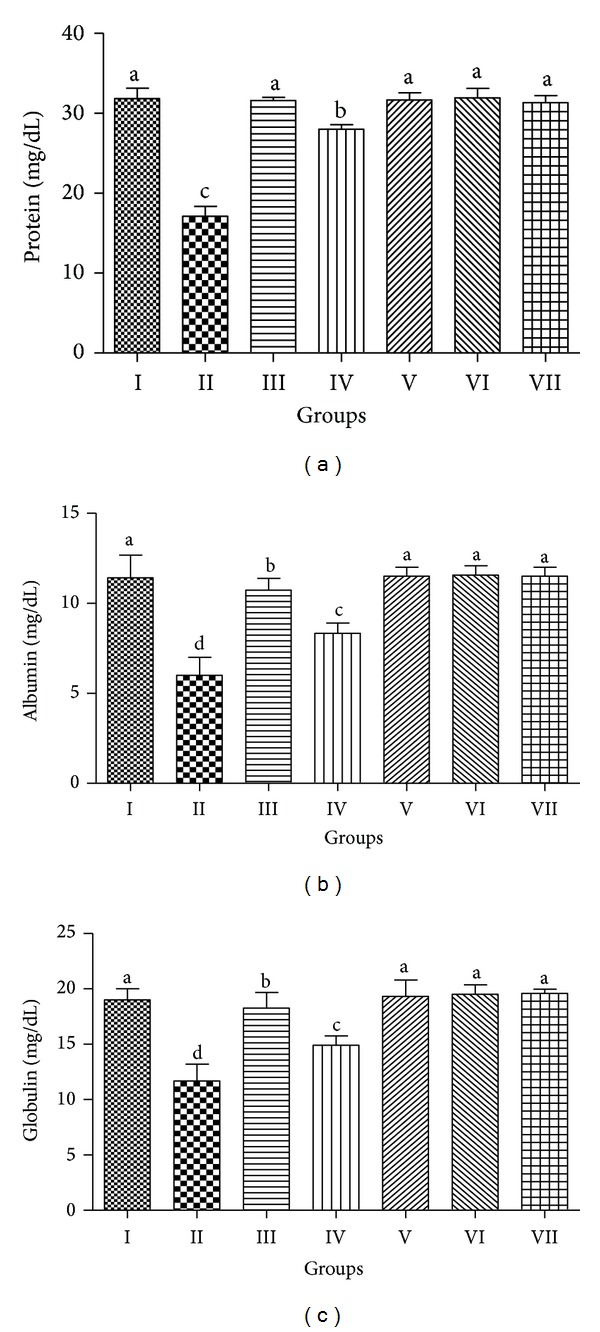
Preventive effect of PCEB on serum (a) protein, (b) albumin, and (c) globulin; mean ± SD (*n* = 6). Means with different letters (a–d) indicate significance at *P* < 0.05; I: control; II: CCl_4_ 1 mL/kg b.w.; III: CCl_4_+Silymarin; IV: CCl_4_+PCEB 200 mg/kg b.w.; V: CCl_4_+PCEB 400 mg/kg b.w.; VI: PCEB 400 mg/kg b.w.; VII: vehicle control.

CCl_4_ treatment in rats caused significant change in the serum protein profile. To analyze the protective effect of PCEB, the fluctuations in serum total protein, albumin, and globulin were analyzed (Figure 1). A significant difference was observed in CCl_4_ treated group in comparison to control group. PCEB treatment showed protective role by significantly (*P* < 0.05) increasing its level in the serum.

#### 3.3.2. Estimation of Haematological Parameters

The protective effect of PCEB on total leukocyte count, neutrophils, lymphocytes, eosinophils, monocytes, and haemoglobin levels against CCl_4_ induced toxicity is shown in Table 2. A significant (*P* < 0.05) decrease in total leukocyte count, neutrophils, and hemoglobin level was observed in CCl_4_ treated rats while the number of lymphocytes, eosinophils, and monocytes significantly increased due to CCl_4_ intoxication. Toxicity of CCl_4_ was ameliorated with cotreatment of PCEB which significantly increased the total leukocyte count and neutrophils and hemoglobin level while decreased the number of lymphocytes, eosinophils, and monocytes in a dose dependent manner.

### 3.4. Pulmonary Toxicity

In this study, protective effect of PCEB against CCl_4_ induced pulmonary toxicity was estimated. In order to characterize the protective effect of PCEB, change in antioxidant enzyme level was evaluated after CCl_4_ treatment.  Table 3 shows the protective effect of PCEB on lung tissue CAT, POD, and SOD. In comparison to control group, the levels of CAT, POD, and SOD in lung tissues were considerably (*P* < 0.05) decreased after the CCl_4_ treatment. PCEB cotreatment reversed the activity of these enzymes towards normal by increasing their level in a dose dependent way.

The protective effects of PCEB on GST, GSH, and GSH-Px in lung tissue are shown in Table 4. In comparison to control group, the activity of GST, GSH, and GSH-Px was decreased (*P* < 0.05) with CCl_4_ treatment. Toxicity of CCl_4_ on GST, GSH, and GSH-Px was erased with cotreatment of PCEB in a dose dependent manner.

Table 5 shows the change in level of GSR, *γ*-GT, and QR after different treatments. Treatment of CCl_4_ decreased (*P* < 0.05) the level of these enzymes in lung tissue versus the control group of rats. Treatment with PCEB recovered the normal level of these enzymes in a dose dependent manner.

The protective effects of PCEB on protein, TBARS, nitrite content, and H_2_O_2_ against CCl_4_ induced alterations are shown in Table 6. After CCl_4_ treatment, a significant decrease (*P* < 0.05) in the level of lung protein as well as increase (*P* < 0.05) in the TBARS, nitrite, and H_2_O_2_ content was observed. Ethanol extract of* P. chinensis* bark reversed the level of protein, TBARS, nitrite content, and H_2_O_2_ in lung tissue towards the normal level in a dose dependent manner.

**Figure 2:**
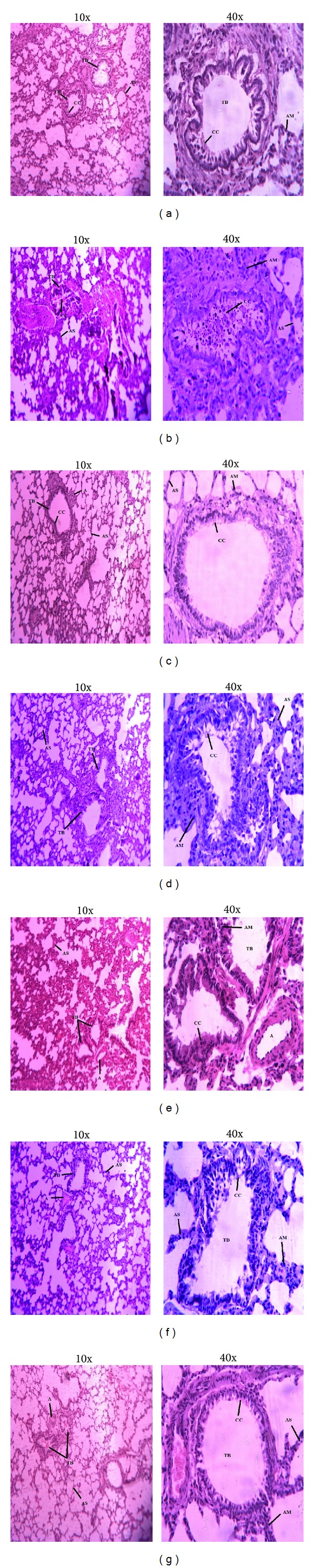
Microphotograph of rat lungs (H & E stain). (a) Control group; (b) CCl_4_ group; (c) Silymarin+CCl_4_ group; (d) PCEB (200 mg/kg b.w.) + CCl_4_ group; (e) PCEB (400 mg/kg b.w.) + CCl_4_ group; (f) PCEB (400 mg/kg b.w.); (g) vehicle control group. TB: terminal bronchiole; CC: Clara cells; AS: alveolar septum; AM: alveolar macrophages; A: arteriole.

It has been proved that lungs can be damaged by administration or ingestion of drugs and chemicals, but it can also be damaged by the environmental toxicant primarily through inhalation. After hematoxylin and eosin staining, various histological features of lung tissue of all experimental groups were observed as shown in Figure 2. Control and vehicle control group showed normal cellular structure and normal morphology. Thin walled alveoli and distinct alveolar septa were observed and junctions of alveolar walls and fibroblasts were also noticeable. The shape of terminal bronchiole was normal and Clara cells were pointed towards the cavity of bronchiole. A clear difference in histology was observed in lung tissue of CCl_4_ treated group. Various injuries like rupture of alveolar walls and bronchioles, aggregation of fibroblasts, and disorganized Clara cells were also observed. Terminal bronchiole had constricted inner epithelium and reduced lumen, and ultimately shortened air passage. The lung sections of rats treated with PCEB showed somewhat normal histological morphology in a dose dependent manner with high dose (400 mg/kg b.w.) showing more protective effect than the low dose (200 mg/kg b.w.). The structure of terminal bronchioles was quite normal but inner epithelium was slightly constricted showing minor effects of CCl_4_. Clara cells were normal and organized. The fibroblasts were also normal, thus showing pulmonary protection effect of PCEB. The Silymarin+CCl_4_ group had reduced the toxic effects of CCl_4_ and reversed the histopathology towards the control group showing quite normal histology with normal alveoli and terminal bronchiole. Normal shaped Clara cells were also observed.

### 3.5. Thyroid Toxicity

**Figure 3:**
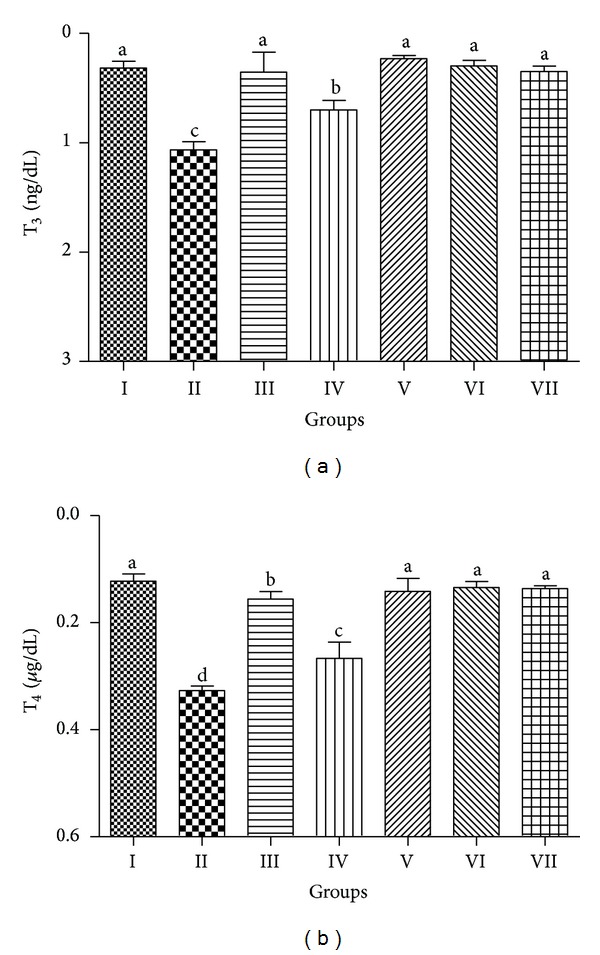
Protective effect of PCEB on thyroid hormones: (a) T_3_ and (b) T_4_; mean ± SD (*n* = 6). Means with different letters (a–d) indicate significance at *P* < 0.05, I: control; II: CCl_4_ 1 mL/kg b.w.; III: CCl_4_+Silymarin; IV: CCl_4_+PCEB 200 mg/kg b.w.; V: CCl_4_+PCEB 400 mg/kg b.w.; VI: PCEB 400 mg/kg b.w.; VII: vehicle control.

**Figure 4:**
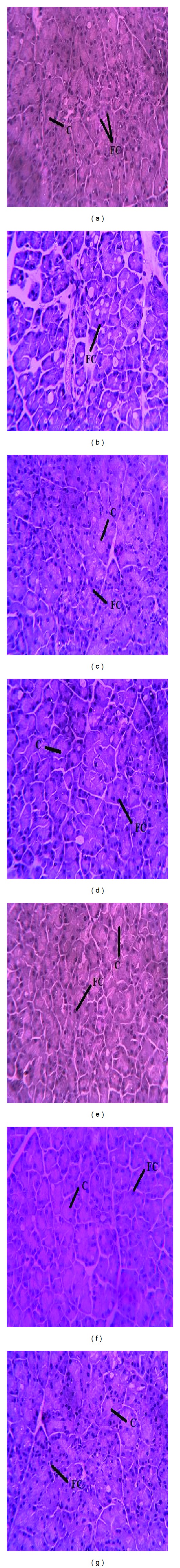
Microphotograph of rat thyroid (H & E stain) at 40x. (a) Representative section of thyroid from the control group showing normal histology with normal shaped follicles cells containing follicles; (b) CCl_4_ group; (c) Silymarin+CCl_4_ group; (d) PCEB (200 mg/kg b.w.) + CCl_4_ group; (e) PCEB (400 mg/kg b.w.) + CCl_4_ group; (f) Only PCEB; (g) vehicle control group. C: Colloid; FC: follicular cells.

Thyroid gland was also examined for CCl_4_ toxicity and PCEB protection against oxidative stress.  Figure 3 depicts the protective effect of PCEB on thyroid hormones, that is, T_3_ and T_4_. CCl_4_ intoxication decreased (*P* < 0.05) the level of T_3_ and T_4_. However, PCEB cotreatment restored the normal level of T_3_ and T_4_ in a dose dependent manner. Normal histology of thyroid tissue with oval and round thyroid follicle was observed in the control group (Figure 4). The lumen of follicles was filled with colloid, lined by a cubical to columnar epithelial cells. Blood vessels were also in normal shape. Hypertrophy and colloid depletion in thyroid follicle were observed in CCl_4_ treated rats. Hyperplasia in follicles was prominent due to change in shape of follicles and congestion of blood vessels. Normal histology was observed in silymarin treated group. Coadministration of PCEB significantly protected thyroid tissue from CCl_4_ injuries in a dose dependent manner but a mild congestion in blood vessels was observed, showing the effects of CCl_4_ toxicity.

## 4. Discussion

Regardless of the enormous advancement in the field of pharmacology and conventional chemistry in creating effective medicines, still plant kingdom is a reservoir of natural therapeutics and offers a valuable source of novel drugs and medicinal entities. Medicinal plants and their phytochemicals are the main source of herbal drugs that can affect the physiological system of animals either directly or indirectly. Plant-based medicines have minimal or no side effects; therefore, these medicines are acknowledged for treatment of number of diseases. Various plants derived antioxidant-based therapeutic medicines are being in use for the prevention and cure of many diseases such as Alzheimer's disease, diabetes, stroke, atherosclerosis, and cancer [27]. The current study mainly concentrates on the role of* P. chinensis* in ameliorating the CCl_4_ induced oxidative stress in rats. It is evident from the results that* P. chinensis* ethanol extract possess protective action against lung and thyroid injuries induced by carbon tetrachloride induced oxidative stress. Results suggest that the extract was effective in dose-dependent manner and maximum protective activity of the extract was obtained when administered at the dose of 400 mg/kg body weight.

Quantitative pharmacological screening exhibited high amount of total phenolic and flavonoid contents. Similar results were obtained by Shah et al. [14] in phytochemical analysis of* S. cordata.*

The screening of bioactive substances present in the plants is the most important job in pharmaceutical research. For this purpose, chromatographic study of plant extracts proved to be very reliable and useful. The TLC of PCEB confirmed the presence of different important phenolic compounds. Decrease in the level of serum protein, albumin, and globulin was observed after the CCl_4_ treatment in rats. The oxidative damage of some amino acids is considered as the major cause of metabolic dysfunction in CCl_4_ induced damage [28]. PCBE at 400 mg/kg ameliorated the synthesis of proteins, albumin, and globulin.

In the current study, CCl_4_ administration greatly affected haematological parameters. A decrease in neutrophils and total leukocyte count was observed which might be due to leucopoenia. The depletion in haemoglobin level in current study could be attributed to destruction of red blood cells, enhanced removal from circulation or decrease in their formation, and disturbed hematopoiesis. Ballinger [29] reported that reduction in haemoglobin level results in iron deficiency anaemia which is characterized by a microcytic hypochromic blood picture. The blood picture was improved in rats cotreated with PCEB, which points towards the protective role of* P. chinensis* against CCl_4_ induced microcytic hypochromic anaemia. These results suggest that ethanol bark extract of* P. chinensis* significantly protected the destructive effects of CCl_4_ in the treated rats.

Various studies have confirmed that free radicals are involved in various metabolic alterations and diseases. Radical reactions are mainly responsible for the* in vivo* toxic effects of CCl_4_. Due to the bioactivation of CCl_4_, various free radicals are produced including CCl_3_OO^•^ and CCl_3_O^•^ radicals, which induce lipid peroxidation and decrease the activities of endogenous antioxidant enzymes, ultimately resulting in organ damage [30]. CCl_4_ treatment causes injuries in liver, kidney, and lung tissue in experimental animals. According to Khan [31], reactive oxygen species produced from CCl_4_ cause oxidative damages in lungs of rats, possibly by altering the antioxidant enzymes status. Various antioxidant enzymes for example, catalase, peroxidase, and superoxide dismutase, play an important role in protecting lung tissues from free radical induced damages [32]. In the present study, the decrease in level of antioxidant enzymes and GSH in lung and thyroid tissues was observed in CCl_4_ treated group. Coadministration of PCEB ameliorated the level of antioxidant enzymes in a dose dependent manner, suggesting protective role of antioxidant enzymes against CCl_4_ generated free radicals. Similar results were observed by Shah et al. [14] on antioxidant enzymes of liver.

Glutathione system is involved in the xenobiotic and drug metabolism. For functional and structural maintenance, level of GSH is very important. GSH is an important thiol protein that functions in the catalysis of numerous metabolites and manages the cellular defence system against oxidative stress generated by free radicals. The maintenance of GSH activity in cell is dependent on the level of glutathione reductase and NADH [33]. Significant decrease in activities of GSH was observed in the present study. The decreased activity of glutathione system in lung tissue of CCl_4_ intoxicated rats might be due to the increased lipid peroxidation or inactivation of the antioxidative enzymes. Administration of PCEB ameliorated the CCl_4_ toxicity, thereby increasing the activity of antioxidant enzymes and GSH. Khan [31] reported similar observations during administration of* Launaea procumbens* methanol extract against CCl_4_ induced oxidative stress.* In vivo* studies carried out by other workers also point out that CCl_4_ reduces the level of GSH in lung and thyroid tissues [34]. Similar results were obtained for the silymarin treated group which is a known antioxidant and commonly used as hepatoprotective agent [35]. Thus, activation of these enzymes by the administration of* P. chinensis* bark extract clearly shows that PCEB through its free radical scavenging activity could exert a beneficial action against pathophysiological alterations caused by free radicals.

Treatment of rats with CCl_4_ causes oxidative damage to pulmonary and thyroid proteins and lipids which are the main cause of producing toxicity in humans by CCl_4_ [3]. Lipids confined in the cell membranes are very sensitive to the oxidative stress. Lipid peroxidation converts polyunsaturated fatty acids into small and more reactive elements. CCl_4_ is a toxic chemical which produces various free radicals causing lipid peroxidation. Increase in lipid peroxidation is calculated in terms of thiobarbituric acid reacting substance (TBARS) which measures the damage caused to the membranes by free radicals [36]. So, TBARS level and H_2_O_2_ content are supposed to be main markers of CCl_4_-induced oxidative stress [37]. The nitrite ion is a ligand which binds to the metal centres and causes vasodilation by producing nitric oxide. In the current study, decrease in protein content was observed while TBARS level, nitrite, and H_2_O_2_ increased due to CCl_4_ treatment which was restored to normal level after treating with PCEB. Oxidative stress induced by CCl_4_ was also evident on lung and thyroid histomorphological level. In current study PCEB reduced the CCl_4_ induced toxicity in lung tissues as shown by normal structure of terminal bronchioles and Clara cells while marked reduction in interstitial infiltration was seen.* In vivo* infection and inflammation in the lung tissue of CCl_4_ treated rats show the deleterious effects of CCl_4_. Edema and inflammatory responses of lungs are positively linked with the function of lungs, together with the oxygenation index and the airway pressure [38]. Lung injuries and inflammation can be attenuated by inhibiting the production of ROS. PCEB treated rats showed marked reduction in epithelial degeneration and decrease in number of alveolar macrophages with suppression of inflammatory cellular infiltration. The protective effect of PCEB might be due to the presence of tannin in the extract, as it has been reported to have anti-inflammatory properties [39].

In the current study, the* in vivo* protective effect of PCEB against CCl_4_ induced thyroid toxicity was also investigated. Free radicals produced by bioactivation of CCl_4_ increase lipid peroxidation and decrease the level of antioxidant enzymes resulting in damage to thyroid tissue. In this study, CCl_4_ treatment caused hypothyroidism as evident by the decrease in level of serum T_3_ and T_4_. T_3_ and T_4_ are the two main hormones of the thyroid gland and are continuously required by body for normal growth. Decrease in level of these hormones depicts malfunctioning of thyroid gland. PCEB treatment ameliorated the CCl_4_ induced toxicity and increased the level of T_3_ and T_4_ in serum of rats. Our results are partially consistent with other reports where decrease in T_3_ was recorded, while T_4_ level was not significantly changed [40]. However, other studies are completely comparable to these findings where treatment of rats with CCl_4_ and allyl alcohol significantly decreased T_3_ and T_4_ level [41, 42].

## 5. Conclusions

It can be concluded that* P. chinensis* bark extract contains antioxidant activity as it prevented the oxidative stress and increased the antioxidant effect in lungs and thyroid tissues of male rats. Our results demonstrate the PCEB protective role against CCl_4_ generated free radicals damages and suggest for further study to isolate the bioactive component in pure form from* P. chinensis* bark.

## Figures and Tables

**Table 1:** TLC of bark ethanol extract of *P. chinensis*.

Color	*R*_*f*_ value	Compound
Dark brown	0.18	Unknown
Purple blue	0.43	Unknown
Yellow	0.57	Rutin
Light blue	0.78	Tannin
Fluorescent yellow	0.75	Unknown
Fluorescent blue	0.81	Gallic acid

**Table 2:** Protective effect of PCEB on hematological parameters.

Treatment	Leukocyte ×10^3^/mm^3^	Neutrophil ×10^3^/mm^3^	Lymphocyte ×10^3^/mm^3^	Eosinophil ×10^3^/mm^3^	Monocyte ×10^3^/mm^3^	Hemoglobin (mg/dL)
Control	5.3 ± 0.02^a^	61.6 ± 2.1^b^	36.3 ± 1.5^d^	1.5 ± 0.3^d^	1.7 ± 0.01^d^	13.7 ± 0.20^a^
CCl_4_ 1 mL/kg	4.6 ± 0.32^d^	57.3 ± 1.5^d^	48.6 ± 3.1^a^	2.6 ± 0.2^a^	2.8 ± 0.10^a^	11.9 ± 0.15^d^
CCl_4_+Silymarin	5.1 ± 0.55^b^	59.5 ± 0.4^c^	37.0 ± 1.0^c^	1.6 ± 0.1^c^	1.8 ± 0.07^c^	13.7 ± 0.40^a^
CCl_4_+PCEB 200 mg/kg	4.9 ± 0.25^c^	67.7 ± 0.2^a^	41.3 ± 1.5^b^	1.9 ± 0.1^b^	2.1 ± 0.32^b^	13.2 ± 0.10^c^
CCl_4_+PCEB 400 mg/kg	5.2 ± 0.47^bc^	65.7 ± 0.2^a^	37.5 ± 1.5^c^	1.7 ± 0.1^bc^	1.9 ± 0.50^c^	13.4 ± 0.20^b^
PCEB 400 mg/kg	5.2 ± 0.17^b^	59.0 ± 1.0^b^	36.9 ± 0.2^d^	1.4 ± 0.1^e^	1.8 ± 0.04^cd^	13.6 ± 0.40^a^
Vehicle control	5.3 ± 0.18^a^	61.2 ± 0.4^b^	35.8 ± 1.0^bd^	1.5 ± 0.1^d^	1.7 ± 0.05^d^	13.5 ± 0.01^ab^

Mean ± SD (*n* = 6). Means with different letters (a–e) indicate significance at *P* < 0.05.

**Table 3:** Protective effect of PCEB on lung CAT, POD, and SOD.

Treatment	CAT (U/min)	POD (U/min)	SOD (U/mg protein)
Control	4.7 ± 0.16^b^	11.1 ± 0.61^c^	3.7 ± 0.18^a^
CCl_4_ 1 mL/kg b.w.	1.1 ± 0.17^d^	3.1 ± 0.28^f^	0.9 ± 0.43^e^
CCl_4_+Silymarin	5.3 ± 0.50^a^	19.4 ± 0.84^a^	3.4 ± 0.10^ab^
CCl_4_+PCEB 200 mg/kg b.w.	2.1 ± 0.15^c^	10.1 ± 0.58^d^	1.4 ± 0.55^d^
CCl_4_+PCEB 400 mg/kg b.w.	5.4 ± 0.53^a^	17.2 ± 0.73^b^	3.1 ± 0.58^bc^
PCEB 400 mg/kg b.w.	4.7 ± 0.16^b^	10.5 ± 0.59^cd^	3.2 ± 0.19^bc^
Vehicle control	4.2 ± 0.14^b^	11.0 ± 0.01^c^	3.3 ± 0.25^b^

Mean ± SD (*n* = 6). Means with different letters (a–e) indicate significance at *P* < 0.05.

**Table 4:** Protective effect of PCEB on lung GST, GSH, and GSH-Px.

Treatment	GST (nM/min/mg protein)	GSH (nM/min/mg protein)	GSH-Px (nM/min/mg protein)
Control	177.0 ± 2.9^c^	21.7 ± 1.31^a^	144.3 ± 2.78^a^
CCl_4_ 1 mL/kg b.w.	94.3 ± 4.3^f^	14.8 ± 1.25^c^	78.7 ± 3.44^e^
CCl_4_+Silymarin	206.7 ± 6.5^a^	18.5 ± 1.11^b^	137.7 ± 4.29^b^
CCl_4_+PCEB 200 mg/kg b.w.	163.0 ± 4.6^d^	16.8 ± 1.25^bc^	115.3 ± 4.65^d^
CCl_4_+PCEB 400 mg/kg b.w.	189.3 ± 5.0^b^	17.2 ± 1.24^b^	120.3 ± 5.82^c^
PCEB 400 mg/kg b.w.	169.0 ± 6.3^c^	20.8 ± 1.10^a^	137.0 ± 1.34^b^
Vehicle control	171.4 ± 2.7^c^	21.3 ± 1.12^a^	141.5 ± 1.36^a^

Mean ± SD (*n* = 6). Means with different letters (a–e) indicate significance at *P* < 0.05.

**Table 5:** Protective effect of PCEB on lung *γ*-GT, GSR, and QR.

Treatment	*γ*-GT (nM/min/mg protein)	GSR (nM/min/mg protein)	QR (nM/min/mg protein)
Control	402.7 ± 10.3^a^	233.7 ± 6.3^b^	217.5 ± 4.0^a^
CCl_4_ 1 mL/kg b.w.	102.0 ± 5.5^d^	191.7 ± 4.4^e^	129.3 ± 2.0^e^
CCl_4_+Silymarin	375.0 ± 5.6^ab^	215.3 ± 2.3^d^	204.5 ± 1.2^b^
CCl_4_+PCEB 200 mg/kg b.w.	108.7 ± 6.4^d^	176.3 ± 4.4^f^	158.4 ± 4.3^f^
CCl_4_+PCEB 400 mg/kg b.w.	124.6 ± 6.7^d^	244.3 ± 2.2^a^	197.2 ± 2.4^d^
PCEB 400 mg/kg b.w.	325.0 ± 7.6^c^	222.0 ± 3.2^c^	200.4 ± 1.1^c^
Vehicle control	361.0 ± 5.9^bc^	210.9 ± 2.5^d^	209.6 ± 2.3^b^

Mean ± SD (*n* = 6). Means with different letters (a–e) indicate significance at *P* < 0.05.

**Table 6:** Protective effect of PCEB on lung Protein, TBARS, nitrite, and H_2_O_2_.

Treatment	Protein (*μ*g/mg tissue)	TBARS (nM/min/mg protein)	Nitrite (*μ*M/mL)	H_2_O_2_ (nM/min/mg tissue)
Control	2.2 ± 0.16^a^	3.5 ± 0.16^e^	44.9 ± 1.58^e^	1.4 ± 0.17^e^
CCl_4_ 1 mL/kg b.w.	0.8 ± 0.14^e^	6.1 ± 0.18^a^	71.8 ± 2.25^a^	2.9 ± 0.15^a^
CCl_4_+Silymarin	2.1 ± 0.15^c^	3.6 ± 0.35^d^	52.2 ± 1.19^cd^	1.5 ± 0.11^d^
CCl_4_+PCEB 200 mg/kg b.w.	1.6 ± 0.38^d^	4.3 ± 0.15^b^	67.3 ± 0.43^b^	2.3 ± 0.52^b^
CCl_4_+PCEB 400 mg/kg b.w.	2.2 ± 0.15^bc^	3.7 ± 0.36^c^	55.2 ± 0.10^c^	1.6 ± 0.15^c^
PCEB 400 mg/kg b.w.	2.2 ± 0.14^b^	3.6 ± 0.92^e^	53.0 ± 1.66^d^	1.5 ± 0.15^d^
Vehicle control	2.2 ± 0.13^a^	3.6 ± 0.16^e^	46.9 ± 1.15^e^	1.4 ± 0.75^e^

Mean ± SD (*n* = 6). Means with different letters (a–e) indicate significance at *P* < 0.05.
